# Intensification with dipeptidyl peptidase-4 inhibitor, insulin, or thiazolidinediones and risks of all-cause mortality, cardiovascular diseases, and severe hypoglycemia in patients on metformin-sulfonylurea dual therapy: A retrospective cohort study

**DOI:** 10.1371/journal.pmed.1002999

**Published:** 2019-12-26

**Authors:** Carlos K. H. Wong, Kenneth K. C. Man, Margaret Shi, Esther W. Chan, Chu Wa Ho, Emily T. Y. Tse, Ian C. K. Wong, Cindy L. K. Lam

**Affiliations:** 1 Department of Family Medicine and Primary Care, Li Ka Shing Faculty of Medicine, The University of Hong Kong, Hong Kong SAR, China; 2 Centre for Safe Medication Practice and Research, Department of Pharmacology and Pharmacy, Li Ka Shing Faculty of Medicine, The University of Hong Kong, Hong Kong SAR, China; 3 Research Department of Policy and Practice, School of Pharmacy, University College London, London, United Kingdom; Harvard Medical School, UNITED STATES

## Abstract

**Background:**

Although patients with type 2 diabetes mellitus (T2DM) may fail to achieve adequate hemoglobin A1c (HbA1c) control despite metformin-sulfonylurea (Met-SU) dual therapy, a third-line glucose-lowering medication—including dipeptidyl peptidase-4 inhibitor (DPP4i), insulin, or thiazolidinedione (TZD)—can be added to achieve this. However, treatment effects of intensification with the medications on the risk of severe hypoglycemia (SH), cardiovascular disease (CVD), and all-cause mortality are uncertain. Study aim was to compare the risks of all-cause mortality, CVD, and SH among patients with T2DM on Met-SU dual therapy intensified with DPP4i, insulin, or TZD.

**Methods and findings:**

We analyzed a retrospective cohort data of 17,293 patients with T2DM who were free from CVD and on Met-SU dual therapy and who were intensified with DPP4i (*n =* 8,248), insulin (*n =* 6,395), or TZD (*n =* 2,650) from 2006 to 2017. Propensity-score weighting was used to balance out baseline covariates across groups. Hazard ratios (HRs) for all-cause mortality, CVD, and SH were assessed using Cox proportional hazard models. Mean age of all patients was 58.56 ± 11.41 years. All baseline covariates achieved a balance across the 3 groups. Over a mean follow-up period of 34 months with 49,299 person-years, cumulative incidences of all-cause mortality, SH, and CVD were 0.061, 0.119, and 0.074, respectively. Patients intensified with insulin had higher risk of all-cause mortality (HR = 2.648, 95% confidence interval [CI] 2.367–2.963, *p* < 0.001; 2.352, 95% CI 2.123–2.605, *p* < 0.001) than those intensified with TZD and DPP4i, respectively. Insulin users had the greatest risk of SH (HR = 1.198, 95% CI 1.071–1.340, *p* = 0.002; 1.496, 95% CI 1.342–1.668, *p* < 0.001) compared with TZD and DPP4i users, respectively. Comparing between TZDs and DPP4i, TZDs were associated with a higher risk of SH (HR = 1.249, 95% CI 1.099–1.419, *p* < 0.001) but not all-cause mortality (HR = 0.888, 95% CI 0.776–1.016, *p* = 0.084) or CVD (HR = 1.005, 95% CI 0.915–1.104, *p* = 0.925). Limitations of this study included the lack of data regarding lifestyle, drug adherence, time-varying factors, patients’ motivation, and cost considerations. A limited duration of patients intensifying with TZD might also weaken the strength of study results.

**Conclusions:**

Our results indicated that, for patients with T2DM who are on Met-SU dual therapy, the addition of DPP4i was a preferred third-line medication among 3 options, with the lowest risks of mortality and SH and posing no increased risk for CVD events when compared to insulin and TZD. Intensification with insulin had the greatest risk of mortality and SH events.

## Author summary

**Why was this study done?:**

- For those who fail dual therapy of metformin and sulfonylurea (Met-SU) after treatment initiation for 3 months, treatment intensification with a third-line glucose-lowering medication is considered as logical stepwise approach of pharmacotherapy.
- Current guidelines endorsed the addition of thiazolidinedione (TZD), dipeptidyl peptidase‐4 inhibitors (DPP4i’s), or basal insulin regimen as third-line options.
- This study directly compared the risks of cardiovascular disease (CVD) and all-cause mortality among type 2 diabetes mellitus (T2DM) patients receiving DPP4i, TZD, or insulin as a third-line medication.

**What did the researchers do and find?:**

- We analyzed a population-wide retrospective cohort with data collected from the Hong Kong Hospital Authority database from January 2006 to December 2017.
- Patients who were free from CVD and on Met-SU dual therapy, and whose drug treatment was intensified with DPP4i (*n =* 8,248), insulin (*n =* 6,395), or TZD (*n =* 2,650), were included.
- Patients intensified with insulin had higher risk of all-cause mortality than those intensified with TZD and DPP4i, respectively.
- Insulin users had the greatest risk of severe hypoglycemia (SH) compared with TZD and DPP4i users, respectively, whereas TZD users were associated with a higher risk of SH than DPP4i users.

**What do these findings mean?:**

In this population-based retrospective cohort study of patients with T2DM after failure of Met-SU dual therapy, TZD was associated with greater risk of SH than DPP4i, whereas insulin was associated with the greatest risks of all-cause mortality and SH. In this cohort, DPP4i had a lower risk of all-cause mortality compared with insulin, and a lower risk of SH compared with TZD and insulin. Caution needs to be exercised when using TZD or insulin because of its high risk for death or SH.

## Introduction

Type 2 diabetes mellitus (T2DM) is a chronic condition with a potential risk of developing long-term complications without adequate glycemic control [1]. The combined use of glucose-lowering medications in addition to lifestyle modification and monotherapy are often required to achieve personalized glycemic targets in patients with T2DM. For those who had inadequate glycemic control under dual therapy of metformin and sulfonylurea (Met-SU) after treatment initiation for 3 months, treatment intensification with a third-line glucose-lowering medication is considered as logical stepwise approach of pharmacotherapy [2]. The updated Position Statement [3] by the American Diabetes Association (ADA) and the European Association for the Study of Diabetes (EASD) endorsed the addition of one of the following glucose-lowering medications as a third-line option when optimal glycemic control is not achieved after 3 months of dual therapy: thiazolidinedione (TZD), dipeptidyl peptidase‐4 inhibitors (DPP4i), sodium‐glucose co‐transporter‐2 (SGLT2) inhibitors, glucagon‐like peptide‐1 receptor agonists (GLP1-RAs), or basal insulin regimen. This clinical practice has also been recommended in the National Institute for Health and Care Excellence (NICE) guideline in 2015 whereby triple therapy should be considered when dual therapy has not continued to control hemoglobin A1c (HbA1c) to below an individual’s target [4]. The consensus report published in 2018 by the ADA and the EASD [5], which was reinstated in 2019 by the ADA [6], advocated the use of TZD following Met-SU therapy when cost is a major issue in patients without existing cardiovascular disease (CVD). Furthermore, the cost-effectiveness of third-line medications was assessed by the Canadian Agency for Drugs and Technologies in Health in 2013 [7]. The addition of basal insulin to Met-SU therapy was the most favorable treatment regimen in terms of cost-effectiveness estimates [7]. Nevertheless, patient preferences, risks and benefits of medications, age, initial HbA1c level, and other patient-specific factors are determinants of decisions about third-line drug options [8].

Previous literature [9,10] has shown that DPP4i’s were not associated with cardiovascular adverse effects and can be used safely even in the elderly. Nevertheless, caution needs be exercised when prescribing saxagliptin (DPP4i) and alogliptin (DPP4i) to patients who are at high risk of heart failure [3,9,11,12]. The TZD class is highly effective in reducing HbA1c level and is associated with a low risk of hypoglycemia [12]. Despite the risk of fluid retention with the likelihood of heart failure, pioglitazone (TZD) has been shown to confer cardiovascular benefits, and it can be given in a moderate dose (≤30 mg) for side effects to be mitigated [12]. On the other hand, rosiglitazone (TZD) was subsequently suspended from the European market because of its associated high risk of myocardial infarction and heart failure [3,11]. With regard to insulin, a vicious cycle is likely to be present between persistently high HbA1c level and insulin resistance, whereby an increased dose is necessary to control hyperglycemia leading to further weight gain, resulting in a further increase in CVD risk [11]. Compared to DPP4i, insulin was reported to have a higher risk of CVD and all-cause mortality [10]. Hypoglycemia is a known risk factor for cardiovascular adverse events, and the use of insulin may be linked to an increase in life-threatening hypoglycemia events requiring external assistance for recovery [13].

Several network meta-analyses [13–15] of randomized controlled trials (RCTs) with a duration of pharmacotherapy of less than 1 year synthesized the short-term intermediate outcomes of each third-line drug option in patients with dual therapy failure. Review of the RCTs with treatment duration between 6 and 12 months demonstrated that the addition of a third-line therapy to dual therapy resulted in improvement in HbA1c from −0.56% to −0.94% and was associated with greater risks of hypoglycemia [16]. Furthermore, treatment intensification with DPP4i to Met-SU dual therapy resulted in significantly less weight gain compared with adding insulin or TZD as the third-line medication [14,17]. However, previous analyses lacked large-scale long-term follow-up regarding the impact of third-line drug options on CVD and mortality outcomes. Notably, when DPP4i was added to Met-SU dual therapy, additional cardiovascular benefits with a lower risk of all-cause mortality were demonstrated in a nationwide retrospective cohort study, in comparison to other oral glucose-lowering medications (i.e., acarbose, meglitidine) [18]. In addition, those on DPP4i as a third-line treatment had a significantly lower risk of heart failure compared with those on a meglitidine add-on regimen. However, no statistically significant difference was seen in stroke risk reduction compared with TZD [18].

The present study examined the treatment effect of intensification with DPP4i, insulin, or TZD on the risk of severe hypoglycemia (SH), CVD, and all-cause mortality among patients with T2DM on Met-SU dual therapy. We hypothesized that patients receiving DPP4i as triple therapy would have lower risks of all-cause mortality, SH, and CVD compared with those receiving insulin or TZD. Moreover, it was hypothesized that intensification with insulin as a third-line medication would be most detrimental to outcome events among the 3 medications.

## Methods

Ethics approval of this study was granted by the Institutional Review Board of the University of Hong Kong/Hospital Authority Hong Kong West Cluster (Reference No. UW 16–1018).

### Data source description

We analyzed the population-based retrospective cohort from the Hong Kong Hospital Authority administrative database in the Hong Kong adult diabetes population from January 1, 2006, to December 31, 2017. The Hospital Authority database has been extensively used for conducting high-quality large population-based studies [19–22]. Documented diabetes mellitus diagnosis was defined as the International Classification of Primary Care, Version 2 (ICPC‐2) codes T89/T90 or International Statistical Classification of Diseases and Related Health Problems, 9th Revision, Clinical Modification (ICD‐9‐CM) codes 250.x. The database contains comprehensive individual patient-level information on prescription and dispensing of glucose-lowering medication, serial readings of anthropometric and laboratory variables, and presence of comorbidities as classified based on the ICD‐9‐CM or ICPC-2 diagnosis codes. A prospective study protocol or analysis plan was not available when designing this study. This study’s statistical analysis plan was developed in March 2019. Changes in the analysis, including those sensitivity and additional analyses made in response to reviewers’ comments, took place in October 2019.

### Identification of study population

All glucose-lowering medication dispensed in pharmacy departments managed under the Hospital Authority during the study period were identified for this study. Information on the date of drug prescribing and dispensing, dosage unit, and quantity was recorded. We included patients who were on dual therapy of Met-SU in the study period and were subsequently intensified with one of the following glucose-lowering medications: DPP4i, insulin, or TZD. Patients were excluded who met the following criteria: less than 18 years old, had type 1 diabetes mellitus, had no diabetes mellitus diagnosis code, had a CVD event occur before the initiation of the third-line medication, received first glucose-lowering medication before 2007 (to allow 1-year window period), received other glucose-lowering medications within 180 days after third-line initiation, and received other glucose-lowering medication drug classes before Met-SU was commenced. Baseline date of eligible patients was defined as the date of initiating third-line medication. Patients were observed from the baseline date until the occurrence of study outcome, death from any cause, or being censored at the last follow-up date, whichever came first.

### Outcome measures

Our study outcomes were all-cause mortality, SH, and composite CVD (acute myocardial infarction, other ischemic heart disease, congestive heart failure, stroke, and peripheral vascular disease). Both the SH and CVD events were identified by the diagnosis codes of the ICD-9-CM and the ICPC-2. All the ICD‐9‐CM and ICPC-2 diagnosis codes for comorbidities and event outcomes are listed in S1 Table.

### Baseline covariates

The baseline covariates included age, gender, and clinical characteristics such as body weight, body mass index (BMI), HbA1c, systolic blood pressure (SBP), diastolic blood pressure (DBP), total cholesterol levels, low-density lipoprotein cholesterol (LDL-C), high-density lipoprotein cholesterol (HDL-C), Charlson comorbidity index (CCI), history of SH, and duration of diabetes mellitus drug dispensed before intensification with third-line medication (i.e., the time between the first glucose-lowering medication prescription and the first third-line medication prescription).

### Statistical analysis

Patients were grouped according to their third-line medication. Baseline characteristics were described respectively (mean ± SD for continuous variables, *N* [%] for categorical variables).

To address missing baseline data, multiple imputation by chained equations (MICE) [23] was used for 3 groups. BMI, HbA1c, SBP, DBP, LDL-C, total cholesterol, HDL-C, serum creatinine, triglyceride, and fasting glucose were imputed by other parameters such as gender, age, duration between first-line medication and third-line medication, history of SH, and CCI. Model parameters were estimated from multiple imputed data and then used to obtain multiple-imputation linear predictions by applying Rubin’s combination rules observation wise to the completed-data predictions [23]. Propensity-score weighting was applied using the predictions obtained after MICE.

To minimize the outcome bias due to discrepancy in baseline covariates, inverse probability of treatment weights (IPTW) using the propensity score was applied to balance covariates across the 3 groups. A multinomial logistics regression model was performed to calculate the propensity scores of each patient in group and included the covariates of age, gender, CCI, and duration between first-line medication and third-line medication. Duration of patient on Met-SU dual therapy was calculated to account for immortal time bias [24] and immortal person-time exposed to Met-SU in each group. The IPTW using the propensity scores was implemented using a user-written command marginal mean weighting through stratification. The lowest and highest 1% (corresponding to the 1st and 99th percentiles) propensity-score weights in each group were removed to trim extreme weights [25]. By repeating these steps 5 times, propensity-score weightings and treatment effects estimated from each imputed dataset were combined to obtain overall estimates of treatment effect. In the context of IPTW, the multiple imputation followed by pooling treatment effects estimates across imputed datasets is the preferred approach [26]. After the propensity-score weighting, the balance of baseline covariates between the groups was further assessed using the absolute standardized mean difference (ASMD). All maximum pairwise ASMDs less than 0.2 implied optimal balance between the groups [27].

Incidence rates (IRs) of each outcome event for each group were estimated using the total number of patients with event occurrence during the follow-up period divided by person-years at risk. A Cox proportional hazards regression model was used to examine the association between the third-line medications and incidence of events. Hazard ratios (HRs) and 95% confidence intervals (CIs) were reported for each treatment group in the regression model. A log-rank test was used to compare the equality of the survival curves between the groups. Predictive accuracy of Cox models was assessed and compared using Harrell’s discrimination C-index, ranging from 0 to 1. A value of 0.5 indicates no predictive discrimination, and values of 0 or 1.0 indicate perfect separation of patients. Proportional hazards assumptions were confirmed through the Schoenfeld residuals test. Goodness of fit of the Cox regression model was assessed using the Akaike information criterion and Bayesian information criterion.

A sensitivity analysis was conducted to include pioglitazone only in the TZD drug class and exclude rosiglitazone because it had already been taken off the market in many countries. Likewise, the effects of basal insulin (Neutral Protamine Hagedorn [NPH] insulin and long-acting insulin) within the insulin class in lowering the risks of all-cause mortality, SH, and CVD were assessed in a sensitivity analysis. The competing risk for mortality was accounted for by the analysis of SH and CVD events, and complete-case analysis in sensitivity analyses. We calculated the E-values as a sensitivity analysis to quantify the potential for unmeasured confounding bias on observed treatment–outcome association [28,29]. The E-value is the minimum strength of association required between an unmeasured confounder and treatment and between confounder and outcome—conditional on measured covariates—to negate the observed treatment-outcome association [28,29]. E-values for each outcome were calculated using an online calculator [30].

All statistical analyses were performed using STATA version 13.0 (StataCorp LP, College Station, Texas). All significance tests were two‐tailed, and *p* < 0.05 was taken to indicate statistical significance. Statistical analyses were conducted by two coauthors (CKHW and CWH) and cross-checked for quality assurance. ​

## Results

**Fig 1:**
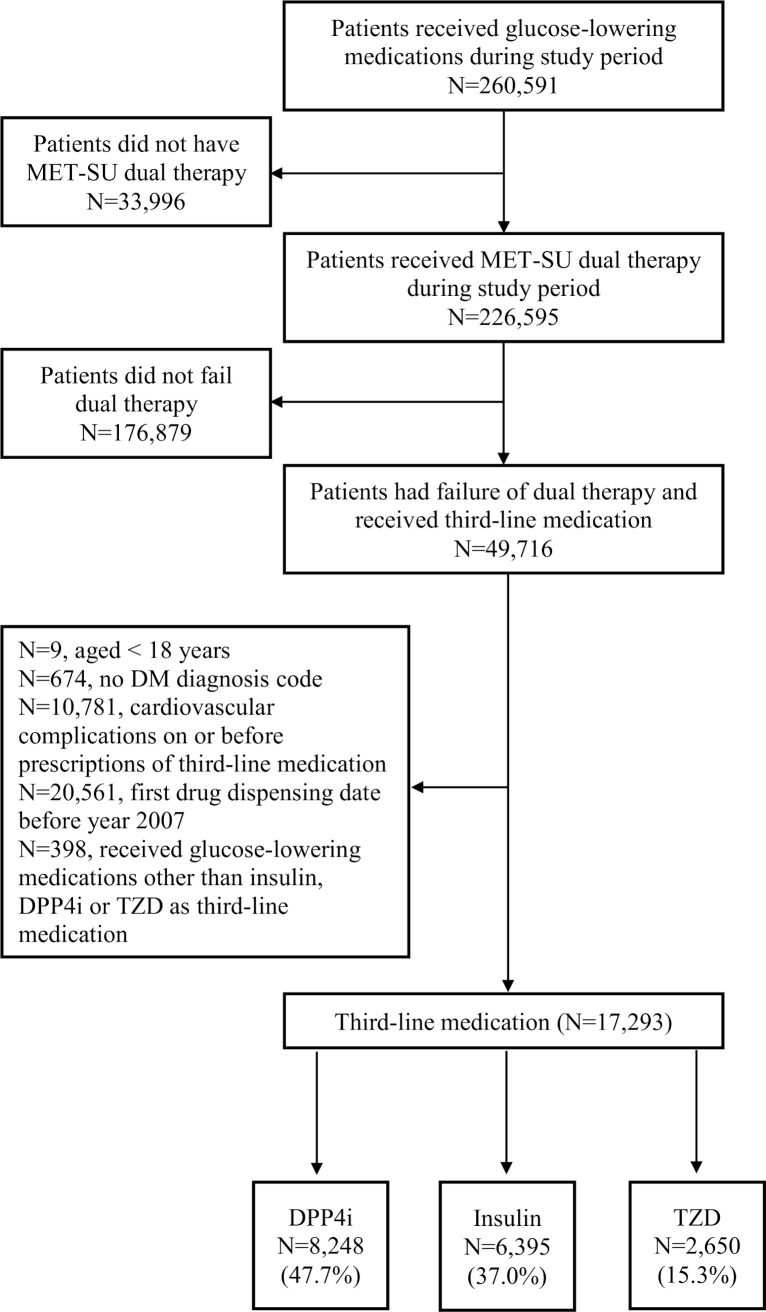
Enrolment of patients who had failure of Met-SU dual therapy and received DPP4i, insulin, or TZD as third-line medications. DM, diabetes mellitus; DPP4i, dipeptidyl peptidase-4 inhibitor; Met-SU, metformin-sulfonylurea; TZD, thiazolidinedione.

The selection process of the cohort group is outlined in the flowchart in Fig 1. In total, 17,293 eligible patients were included in the current analysis. Among all patients, a majority (47.7%) received DPP4i as their third-line medication, followed by insulin (37.0%) and TZD (15.3%).

### Patient characteristics

Table 1 illustrates the baseline characteristics of patients according to their treatment groups before weighting. All the maximum pairwise ASMDs of the characteristics were less than 0.2 after weighting, implying that all covariates achieved a balance in baseline covariates across the 3 groups. In this cohort, the mean age of all participants was 58.56 years, and mean BMI was 28.45 kg/m^2^. Mean values of SBP and DBP were 132.48 and 76.89 mmHg, respectively. Details of baseline characteristics in each group after weighting are listed in S2 Table.

**Table 1:** Baseline characteristics of patients intensifying with DPP4i, insulin or TZD, as third-line medication.

					Maximum pairwise ASMD	
	Total	DPP4i	Insulin	TZD	Before weighting	After weighting
**General information**						
Total number of participants	17,293	8,248	6,395	2,650		
Age (years), mean ± SD	58.56 ± 11.41	59.22 ± 10.98	58.05 ± 12.27	57.73 ± 10.38	0.138	0.072
Gender, *n* (%)					0.021	0.021
Female	7,959 (46.02%)	3,812 (46.22%)	2,950 (46.13%)	1,197 (45.17%)		
Male	9,334 (53.98%)	4,436 (53.78%)	3,445 (53.87%)	1,453 (54.83%)		
**Clinical parameter**						
Laboratory result, mean ± SD						
HbA1c, %	8.71 ± 1.59	8.49 ± 1.25	9.16 ± 1.97	8.31 ± 1.22	0.476*	0.069
SBP, mmHg	132.48 ± 16.07	132.85 ± 15.59	133.10 ± 17.22	129.84 ± 14.34	0.199	0.021
DBP, mmHg	76.89 ± 9.67	76.96 ± 9.78	76.97 ± 9.87	76.43 ± 8.76	0.057	0.016
LDL-C, mmol/L	2.42 ± 0.77	2.42 ± 0.74	2.49 ± 0.83	2.25 ± 0.68	0.314*	0.097
HDL-C, mmol/L	1.13 ± 0.29	1.13 ± 0.27	1.14 ± 0.31	1.12 ± 0.27	0.075	0.028
BMI, kg/m^2^	28.45 ± 4.06	28.60 ± 4.04	28.15 ± 4.10	28.73 ± 4.01	0.142	0.032
Waist, cm	96.10 ± 19.15	95.99 ± 9.85	96.07 ± 24.58	96.50 ± 25.54	0.033	0.076
TC, mmol/L	4.38 ± 0.94	4.37 ± 0.89	4.48 ± 1.02	4.15 ± 0.84	0.340*	0.101
Triglyceride, mmol/L	1.87 ± 1.30	1.86 ± 1.24	1.93 ± 1.42	1.78 ± 1.20	0.113	0.054
Creatinine (serum), μmol/L	89.04 ± 67.67	85.02 ± 51.97	98.36 ± 92.28	79.05 ± 23.79	0.246*	0.054
eGFR, mL/min/1.73 m^2^	86.09 ± 28.82	86.84 ± 27.52	84.48 ± 32.80	87.65 ± 21.40	0.106	0.095
Fasting glucose, mmol/L	9.60 ± 3.02	9.36 ± 2.63	10.18 ± 3.53	8.96 ± 2.57	0.373*	0.084
Prior severe hypoglycemia, n (%)	1,299 (7.51%)	456 (5.53%)	741 (11.59%)	102 (3.85%)	0.293*	0.011
Duration between first-line medication and third-line medication (years), mean ± SD	5.54 ± 2.81	5.81 ± 2.71	4.90 ± 2.73	6.26 ± 3.00	0.483*	0.068
Duration of DM (years), mean ± SD	5.43 ± 2.97	5.69 ± 2.87	4.75 ± 2.89	6.36 ± 3.10	0.546*	0.063
Duration of DM (years), *n* (%)					0.309*	0.080
≤5 years	7,210 (46.57%)	2,994 (42.40%)	3,330 (55.66%)	886 (36.34%)		
5–10 years	7,200 (46.50%)	3,579 (50.68%)	2,427 (40.56%)	1,194 (48.97%)		
>10 years	1,073 (6.93%)	489 (6.92%)	226 (3.78%)	358 (14.68%)		
CCI, *n* (%)					0.479*	0.045
1 or 2	2,662 (15.39%)	1,149 (13.93%)	1,112 (17.39%)	401 (15.13%)		
3	4,535 (26.22%)	2,165 (26.25%)	1,520 (23.77%)	850 (32.08%)		
4	4,443 (25.69%)	2,243 (27.19%)	1,386 (21.67%)	814 (30.72%)		
5	2,793 (16.15%)	1,527 (18.51%)	885 (13.84%)	381 (14.38%)		
6 or above	2,860 (16.54%)	1,164 (14.11%)	1,492 (23.33%)	204 (7.70%)		

*Imbalance covariate if maximum pairwise ASMD ≥ 0.2.

**Abbreviations:** ASMD, absolute standardized mean difference; BMI, body mass index; DBP, diastolic blood pressure; DPP4i, dipeptidyl peptidase-4 inhibitor; CCI, Charlson comorbidity index; DM, diabetes mellitus; eGFR, estimated glomerular filtration rate; HbA1c, hemoglobin A1c; HDL-C, high-density lipoprotein cholesterol; LDL-C, low-density lipoprotein cholesterol; SBP, systolic blood pressure; SD, standard derivation; SH, severe hypoglycemia; TC, total cholesterol; TZD, thiazolidinedione

### IRs

Table 2 depicts the cumulative incidence and IRs of all-cause mortality, SH, and CVD across the follow-up period for patients treated with insulin, DPP4i, and TZD as part of the triple therapy. Over a mean follow-up period of 34 months with 49,299 person-years, cumulative incidences of all-cause mortality, CVD, and SH were 0.061, 0.074, and 0.119, respectively. The mean follow-up period of our cohort ranged from 33 to 34 months across outcome events. Upon weighting, patients intensified with insulin had the most incidences of all-cause mortality (IR = 2.748/100 person-years), whereas the patients intensified with TZD had the most incidences of SH (IR = 4.896/100 person-years) and CVD (IR = 2.583/100 person-years).

**Table 2:** Number and incidence rate of all-cause mortality, SH, and CVD events.

	Before weighting							After weighting	
	Cumulative incidence		Crude incidence rate (cases/100 person-years)			Median follow-up periods (months)	Mean follow-up periods (months)	Incidence rate (cases/100 person-years)	
Event	Cases with event	Rate	Estimate	95% CI*	Person-years			Estimate	95% CI*
**Total (*N* = 17,293)**									
All-cause mortality	1,057	0.061	2.144	(2.017–2.277)	49,299.25	28	34	1.805	(1.733–1.878)
SH	2,065	0.119	4.698	(4.497–4.905)	43,959.33	26	33	4.120	(4.006–4.235)
CVD	1,276	0.074	2.698	(2.552–2.850)	47,293.83	26	33	2.445	(2.360–2.532)
**DPP4i as third-line (*N* = 8,248)**									
All-cause mortality	228	0.028	1.035	(0.905–1.178)	22,032.92	29	32	1.120	(1.024–1.222)
SH	688	0.083	3.333	(3.088–3.591)	20,642.92	28	32	3.738	(3.554–3.928)
CVD	477	0.058	2.225	(2.030–2.434)	21,436.42	28	31	2.394	(2.251–2.544)
**Insulin as third-line (*N* = 6,395)**									
All-cause mortality	796	0.124	3.439	(3.204–3.686)	23,147.42	40	43	2.748	(2.609–2.891)
SH	1,236	0.193	6.379	(6.028–6.744)	19,377.25	37	41	3.957	(3.782–4.136)
CVD	732	0.114	3.361	(3.122–3.613)	21,781.92	36	41	2.402	(2.270–2.538)
**TZD as third-line (*N* = 2,650)**									
All-cause mortality	33	0.012	0.801	(0.551–1.125)	4,118.92	13	19	1.200	(1.086–1.323)
SH	141	0.053	3.579	(3.013–4.221)	3,939.17	14	19	4.896	(4.650–5.148)
CVD	67	0.025	1.644	(1.274–2.088)	4,075.50	14	18	2.583	(2.411–2.764)

*The 95% CI was constructed based on Poisson distribution.

**Abbreviations:** CI, confidence interval; CVD, cardiovascular disease; DPP4i, dipeptidyl peptidase-4 inhibitor; SH, severe hypoglycemia; TZD, thiazolidinedione

### Risk of SH, CVD, and all-cause mortality

**Fig 2:**
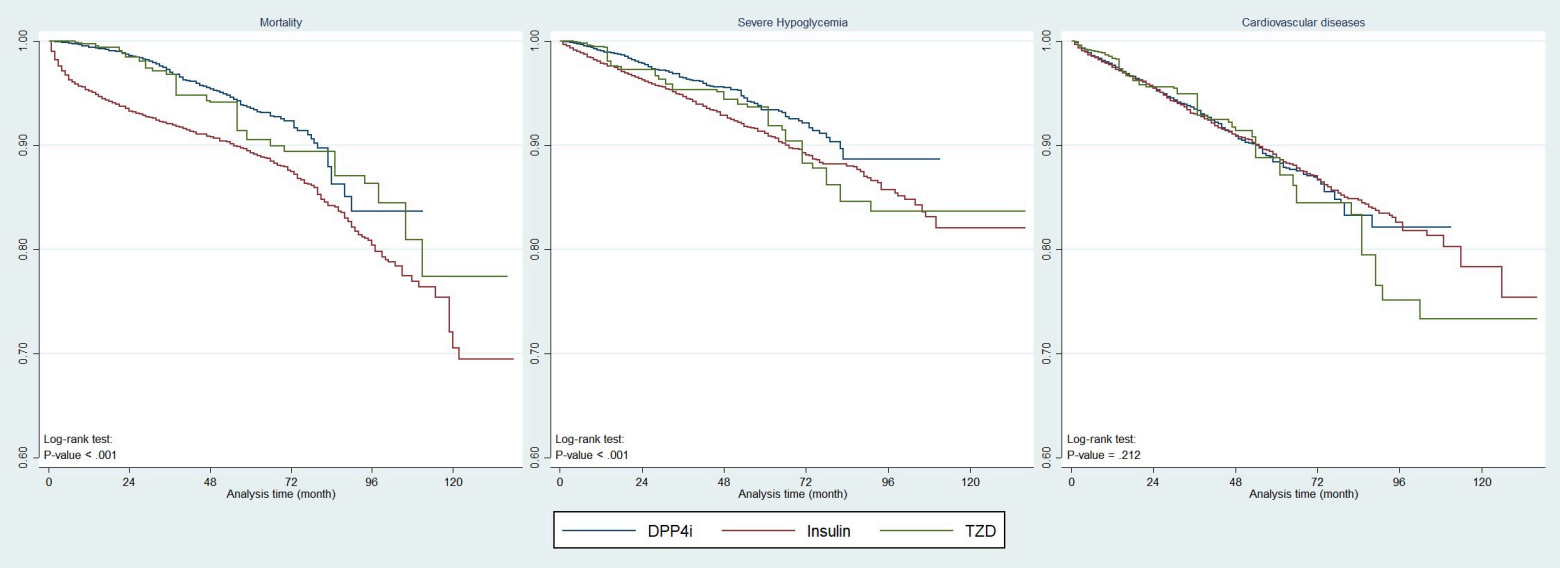
Kaplan Meier survival curves for all-cause mortality, SH, and CVD for T2DM patients with DPP4i, insulin, or TZD as third-line medications after propensity-score weighting. CVD, cardiovascular disease; DPP4i, dipeptidyl peptidase-4 inhibitor; SH, severe hypoglycemia; T2DM, type 2 diabetes mellitus; TZD, thiazolidinedione.

Fig 2 depicts the Kaplan Meier survival curves for all-cause mortality, SH, and CVD events by treatment group.

Table 3 compares the HR of DPP4i, insulin, and TZD against each other. Risk of SH for insulin was increased by about 1.5-fold (HR = 1.496, 95% CI 1.342–1.668, p < 0.001) relative to DPP4i, followed by approximately 1.2-fold (HR = 1.249, 95% CI 1.099–1.419, p < 0.001) for TZD compared to DPP4i. Compared with the risk of SH, the risk of all-cause mortality for insulin was increased further to 2.648 (95% CI 2.367–2.963, p < 0.001) and 2.352 (95% CI 2.123–2.605, p < 0.001) relative to TZD and DPP4i, respectively. On the other hand, the difference in risk of all-cause mortality between TZD and DPP4i was insignificant (HR = 0.888, 95% CI 0.776–1.016, p = 0.084). Similarly, the differences in risk of CVD between insulin and DPP4i (HR = 0.970, 95% CI 0.893–1.053, p = 0.476), between TZD and DPP4i (HR = 1.005, 95% CI 0.915–1.104, p = 0.928), and between insulin and TZD (HR = 0.965, 95% CI 0.883–1.056, p = 0.446) were insignificant. To compare among the 3 medications and other glucose-lowering medications, an additional analysis was conducted (S3 Table). Other glucose-lowering medications showed a significantly higher risk for mortality, SH, and CVD than DPP4i (HR = 1.288, 95% CI 1.154–1.439, p < 0.001; 2.414, 95% CI 2.180–2.674, p < 0.001; 1.834, 95% CI 1.702–1.977, p < 0.001, respectively) and TZD (HR = 1.462, 95% CI 1.301–1.643, p < 0.001; 1.932, 95% CI 1.739–2.146, p < 0.001; 1.889, 95% CI 1.741–2.050, p < 0.001, respectively). When comparing other medications with insulin, the risk of SH and CVD increased while that of mortality decreased. Overall, insulin was associated with the greatest risks in all cause-mortality and SH, followed by TZD and DPP4i, whereas there were no significant differences in risk of CVD across the 3 groups. DPP4i had the lowest risk of all-cause mortality and SH events as a third-line agent for intensification of diabetes management.

**Table 3:** Hazard ratio of all-cause mortality, SH, and CVD events.

Event	TZD (versus DPP4i)			Insulin (versus TZD)			Insulin (versus DPP4i)		
	HR	95% CI	*p*-value	HR	95% CI	*p*-value	HR	95% CI	*p*-value
**All-cause mortality**	0.888	(0.776–1.016)	0.084	2.648	(2.367–2.963)	<0.001*	2.352	(2.123–2.605)	<0.001*
**SH**	1.249	(1.099–1.419)	<0.001*	1.198	(1.071–1.340)	0.002*	1.496	(1.342–1.668)	<0.001*
**CVD**	1.005	(0.915–1.104)	0.928	0.965	(0.883–1.056)	0.446	0.970	(0.893–1.053)	0.476
***Sensitivity analysis*: *Pioglitazone only in TZD drug class***									
Event	**TZD (versus DPP4i)**			**Insulin (versus TZD)**			**Insulin (versus DPP4i)**		
	**HR**	**95% CI**	***p*-value**	**HR**	**95% CI**	***p*-value**	**HR**	**95% CI**	***p*-value**
**All-cause mortality**	0.917	(0.794–1.058)	0.237	2.586	(2.283–2.928)	<0.001*	2.371	(2.140–2.626)	<0.001*
**SH**	1.102	(0.956–1.271)	0.180	1.383	(1.215–1.574)	<0.001*	1.524	(1.367–1.700)	<0.001*
**CVD**	0.925	(0.834–1.026)	0.141	1.080	(0.976–1.195)	0.136	0.999	(0.919–1.085)	0.981
***Sensitivity analysis*: *Basal insulin only in insulin class***									
Event	**TZD (versus DPP4i)**			**Insulin (versus TZD)**			**Insulin (versus DPP4i)**		
	**HR**	**95% CI**	***p*-value**	**HR**	**95% CI**	***p*-value**	**HR**	**95% CI**	***p*-value**
**All-cause mortality**	0.902	(0.791–1.027)	0.120	1.631	(1.461–1.822)	<0.001*	1.471	(1.323–1.635)	<0.001*
**SH**	1.637	(1.456–1.841)	<0.001*	0.830	(0.750–0.918)	<0.001*	1.358	(1.220–1.513)	<0.001*
**CVD**	1.205	(1.103–1.317)	<0.001*	0.839	(0.773–0.912)	<0.001*	1.011	(0.933–1.097)	0.796
***Sensitivity analysis*: *Prandial insulin only in insulin class***									
Event	**TZD (versus DPP4i)**			**Insulin (versus TZD)**			**Insulin (versus DPP4i)**		
	**HR**	**95% CI**	***p*-value**	**HR**	**95% CI**	***p*-value**	**HR**	**95% CI**	***p*-value**
**All-cause mortality**	1.309	(1.147–1.493)	<0.001*	3.250	(2.936–3.598)	<0.001*	4.254	(3.829–4.726)	<0.001*
**SH**	1.620	(1.432–1.832)	<0.001*	1.784	(1.617–1.969)	<0.001*	2.890	(2.604–3.207)	<0.001*
**CVD**	1.312	(1.192–1.443)	<0.001*	1.017	(0.932–1.110)	0.724	1.334	(1.222–1.455)	<0.001*
***Sensitivity analysis*: *Accounting for competing risk of death***									
Event	**TZD (versus DPP4i)**			**Insulin (versus TZD)**			**Insulin (versus DPP4i)**		
	**SHR**	**95% CI**	***p*-value**	**SHR**	**95% CI**	***p*-value**	**SHR**	**95% CI**	***p*-value**
**SH**	1.264	(1.115–1.432)	<0.001*	1.153	(1.031–1.291)	0.013*	1.458	(1.306–1.626)	<0.001*
**CVD**	1.015	(0.926–1.113)	0.758	0.927	(0.849–1.012)	0.092	0.941	(0.866–1.023)	0.155

*Statistically significant at *p* < 0.05.

**Abbreviations:** CI, confidence interval; CVD, cardiovascular disease; DPP4i, dipeptidyl peptidase-4 inhibitor; HR, hazard ratio; SH, severe hypoglycemia; SHR, subdistribution hazard ratio; TZD, thiazolidinedione

### Sensitivity analysis

Given that rosiglitazone and pioglitazone belong to the drug class of TZD, when those who received rosiglitazone medications were excluded in the sensitivity analysis, it became evident that the risk of SH for pioglitazone users becomes insignificant compared with DPP4i (HR = 1.102, 95% CI = 0.956–1.271, *p* = 0.180). In contrast to insulin class, basal insulin has a lower risk of mortality and SH in the comparison group with DPP4i, and a statistically significant lower risk of SH (HR = 0.830, 95% CI 0.750–0.918, *p* < 0.001) and CVD (HR = 0.839, 95% CI 0.773–0.912, *p* < 0.001) than TZD. In the meantime, prandial insulin showed a relatively higher risk for mortality, SH, and CVD compared with DPP4i, and the hazard of CVD was significantly decreased when compared with TZD (HR = 1.017, 95% CI 0.932–1.110, *p* = 0.724) (Table 3). Accounting for the competing risk of death in the CVD outcome analysis, results were in line with those in the main analysis. Insulin was associated with a lower risk of SH compared with TZD (subdistribution hazard ratio [SHR] = 1.153, 95% CI 1.031–1.291, *p* = 0.013) and DPP4i (SHR = 1.458, 95% CI 1.306–1.626, *p* < 0.001) when accounting for the competing risk of death. The E-values as a sensitivity analysis for assessing unmeasured confounding bias were calculated for the HR for all-cause mortality, CVD, and SH outcomes (S4 Table). It was unlikely that an unmeasured or unknown confounder would have greater effect on the outcomes than these known risk factors by having a HR exceeding those E-values (S5 Table).

## Discussion

In more recent years, promising new classes of oral dose forms have become options to add on to the dual combination of Met-SU when required [31]. The present study compared the risks of all-cause mortality, SH, and CVD among patients with T2DM who were on Met-SU dual therapy and intensified with DPP4i, insulin, or TZD. The addition of insulin as a third-line medication was found to have the highest incidence and risk of all-cause mortality, as well as an increased risk of SH. These findings are consistent with our hypothesis that patients with T2DM initiated with insulin as a third-line medication could be at higher risk of SH and all-cause mortality. A possible explanation of an increased mortality rate is the potential that insulin may increase the risk of developing cancer and atherosclerotic vascular diseases due to its atherogenic and mitogenic effects [32]. However, interesting results showed a lower risk of CVD compared with that of TZD. In the meantime, insulin remains an effective, potent glucose-lowering agent with an overall established safety record and is thus considered as part of a combination therapy when hyperglycemia is severe and poorly controlled with the use of oral agents alone [12]. When the use of basal insulin was assessed for the risks of all-cause mortality, SH, and CVD events in sensitivity analysis, basal insulin was shown to be associated with further reduced risks of all outcomes compared to insulin class. Such findings echoed Canadian guidelines that initiation of a single daily dose of insulin NPH under the basal insulin regimen has been recommended since 2013 [7].

DPP4i’s, though ranked the best in number of incidences of all outcomes, were found to be associated with the lowest risk of mortality and SH outcomes. The latest clinical practice guideline “Optimal Use Recommendations for second- and third-line therapy for patients with type 2 diabetes” has recommended that DPP4i be used when patients are unable to use insulin as the third-line medication [7]. It was surprising to see that there was no difference in the risk of CVD between DPP4i and insulin. DPP4i was not associated with a lower risk of CVD than TZD, echoing the notion that DPP4i is known to be non-superior in its cardiovascular protective effects [9]. Notably, among the RCTs of TZD, there was a lack of comparison with other glucose-lowering medications other than with placebos [9]. This confirms the novelty of our study in addressing the research gap in terms of the limited evidence in the area of studying the risks of all-cause mortality, SH, and CVD across third-line medications, specifically DPP4i, insulin, and TZD. Our study further highlighted the importance of a population-based study whereby having a large sample size allows valid conclusions to be drawn for the assessment of risk of all-cause mortality, SH, and CVD for third-line medications for patients with T2DM.

While those using TZD were at greater risk of SH than those using DPP4i (HR = 1.249, 95% CI 1.099–1.419, *p* < 0.001), the risks of all-cause mortality and CVD were not statistically significant. As a subgroup of TZDs, pioglitazone was found to be associated with further decrease in the risk of SH in sensitivity analysis. There is a need for careful monitoring for baseline risks in patients intensifying with TZD and insulin, as well as careful assessment regarding the choice of a new add-on third-line agent for T2DM management.

Collectively, findings of this study provide new insights in understanding the risks of important classes of glucose-lowering agents as third-line medications and prove low risks of all-cause mortality and SH events associated with DPP4i, although insulin and TZD are highly effective glucose-lowering agents. The findings from this study should be considered when making clinical decisions about third-line glucose-lowering medications.

### Limitations

Our study had some limitations. Lifestyle risk factors and issues with drug adherence were not captured in the database and could not be assessed. Therefore, it was not possible to include these factors in the propensity-score weighting. However, the likelihood that unmeasured confounders could affect the treatment–outcome relationship seemed unlikely, as indicated by E-values in sensitivity analysis. In addition, time-varying factors—such as changes in HbA1c, blood pressure, and lipid profile—were also not taken into account in the propensity-score weighting and subsequent multivariable analyses. These could potentially exert an influence on the risk of cardiovascular adverse effects and reduce the validity of results. Moreover, specific individual factors such as a patient’s motivation and cost considerations were not considered; however, propensity-score weighting was applied to other biomedical factors, including age and comorbidities. Furthermore, a limited duration of patients intensifying with TZD could likely have weakened the strength of evidence of the study. Notably, the median length of follow-up varied across different third-line medications. Therefore, it is possible that the risk of events could have been deduced from the data across a limited period of time. Our study results provide an important indication of the relative risks among third-line diabetes medications with respect to all-cause mortality, SH, and CVD. However, since the overall duration of this study was relatively short, additional studies are needed in estimating the long-term effect on cardiovascular events associated with DPP4i as one of the third-line glucose-lowering medications.

This retrospective population-based cohort study evaluated the risks of all-cause mortality, SH, and CVD events for patients intensifying with insulin, DPP4i, or TZD as a third-line medication. Among patients with T2DM on Met-SU dual therapy, DPP4i as a third-line medication had the lowest risk of all-cause mortality and SH events and posed no increased risk for CVD when compared to TZD and insulin. Intensification with insulin as a third-line medication had the greatest risk of mortality and SH events among the 3 glucose-lowering medications. There is a need for careful monitoring for baseline risks in patients intensifying with TZD and insulin, as well as careful assessment regarding the choice of a new third-line medication for T2DM management.

## Supporting information

S1 STROBE Checklist (DOCX)

S1 Table List of relevant diagnosis codes. (DOCX)

S2 Table Baseline characteristics of patient with DPP4i, insulin, or TZD as third-line medication after propensity-score weighting. (DOCX)

S3 Table HR of all-cause mortality, SH, and CVD events for comparisons among DPP4i, insulin, TZD, and other glucose-lowering medications. (DOCX)

S4 Table E-values for outcomes. (DOCX)

S5 Table Adjusted HRs for the associations between the outcomes and observed baseline covariates. (DOCX)

S6 Table HR of all-cause mortality, SH, and CVD events using complete case analysis. (DOCX)

## Abbreviations

ADA: American Diabetes Association
ASMD: absolute standardized mean difference
BMI: body mass index
CCI: Charlson comorbidity index
CI: confidence interval
CVD: cardiovascular disease
DBP: diastolic blood pressure
DPP4i: dipeptidyl peptidase-4 inhibitor
EASD: European Association for the Study of Diabetes
eGFR: estimated glomerular filtration rate
GLP1-RA: glucagon‐like peptide‐1 receptor agonist
HbA1c: hemoglobin A1c
HDL-C: high-density lipoprotein cholesterol
HR: hazard ratio
ICD-9-CM: International Statistical Classification of Diseases and Related Health Problems, 9th Revision, Clinical Modification
ICPC-2: International Classification of Primary Care, Version 2
IR: incidence rate
LDL-C: low-density lipoprotein cholesterol
Met-SU: metformin-sulfonylurea
MICE: multiple imputation by chained equations
NICE: National Institute for Health and Care Excellence
NPH: Neutral Protamine Hagedorn
RCT: randomized controlled trial
SBP: systolic blood pressure
SGLT2: sodium‐glucose co‐transporter‐2
SH: severe hypoglycemia
SHR: subdistribution hazard ratio
T2DM: type 2 diabetes mellitus
TZD: thiazolidinedione
