# Supplementary material for: Intensification with dipeptidyl peptidase-4 inhibitor, insulin, or thiazolidinediones and risks of all-cause mortality, cardiovascular diseases, and severe hypoglycemia in patients on metformin-sulfonylurea dual therapy: A retrospective cohort study
Source: PLoS Med. 2019 Dec 26;16(12):e1002999. doi: 10.1371/journal.pmed.1002999 (PMC6932752; doi:10.1371/journal.pmed.1002999)
Supplement: S1 Table — (DOCX) [file pmed.1002999.s002.docx]

| Supplemental Table 1. List of relevant diagnosis codes | | |  |
| --- | --- | --- | --- |
| Code Type | Description | Diagnosis Code | |
| ICD-9-CM | Hypoglycemia | 250.30-250.33; 250.80-250.83; 251.0-251.2; 270.3; 775.0; 775.6; 962.3 | |
|  | Acute myocardial infarction | 410.00-410.92 | |
|  | Other ischemic heart disease | 411.0; 411.1; 411.81; 411.89; 412; 413.0; 413.1; 413.9; 414.00-414.07; 414.10-414.12; 414.19; 414.2-414.4; 414.8; 414.9 | |
|  | Congestive heart failure | 428.0-428.9 | |
|  | Stroke | 430; 431; 432.0-432.9; 433.00-433.91; 434.00-434.91; 435.0-435.9; 436; 437.0-437.9; 438.0-438.9 | |
|  | Peripheral vascular disease | 250.60-250.69; 440.2; 997.2; 997.6 | |
| ICPC-2 | Acute myocardial infarction | K75 | |
|  | Other ischemic heart disease | K74; K76 | |
|  | Congestive heart failure | K77 | |
|  | Stroke | K89; K90; K91 | |
|  | Peripheral vascular disease | K92 | |
| ICD-10 | Circulatory system disease | I20.9; I21.09; I21.3; I25.1; I25.2; I25.84; I25.9 | |
|  | Hypertensive disease | I11.0; I11.9 | |
|  | Other heart disease | I25.10; I48.91; I50.9; I63.9; I65.23; I65.29; I67.2; I67.9; I73.9 | |
|  | Cardiovascular and ischemic disease | I25.10; I48.91; I50.9; I63.9; I65.23; I65.29; I67.2; I67.9; I73.9 | |
