# Supplementary material for: Intensification with dipeptidyl peptidase-4 inhibitor, insulin, or thiazolidinediones and risks of all-cause mortality, cardiovascular diseases, and severe hypoglycemia in patients on metformin-sulfonylurea dual therapy: A retrospective cohort study
Source: PLoS Med. 2019 Dec 26;16(12):e1002999. doi: 10.1371/journal.pmed.1002999 (PMC6932752; doi:10.1371/journal.pmed.1002999)
Supplement: S2 Table — (DOCX) [file pmed.1002999.s003.docx]

| Supplemental Table 2. Baseline characteristics of patient with DPP4i, insulin or TZD as third-line medication after propensity score weighting | | | | | | | | |
| --- | --- | --- | --- | --- | --- | --- | --- | --- |
| Patient characteristics | After weighting | | | | | | | |
|  |  | (A) | (B) | (C) | (A) vs (B) | (B) vs (C) | (A) vs (C) | Maximum pairwise ASMD |
|  | Total | DPP4i | Insulin | TZD | Pairwise ASMD | Pairwise ASMD | Pairwise ASMD |  |
| **General information** |  |  |  |  |  |  |  |  |
| Total number of participants | NA | | | |  |  |  |  |
| Age (year), Mean ± SD | 58.10 ± 11.46 | 58.45 ± 11.17 | 58.23 ± 11.62 | 57.64 ± 11.57 | 0.019 | 0.051 | 0.072 | 0.072 |
| Gender, % |  |  |  |  | 0.005 | 0.016 | 0.021 | 0.021 |
| Female | 46.99% | 46.52% | 46.73% | 47.70% |  |  |  |  |
| Male | 53.01% | 53.48% | 53.27% | 52.30% |  |  |  |  |
|  |  |  |  |  |  |  |  |  |
| **Clinical parameter** |  |  |  |  |  |  |  |  |
| Laboratory result, Mean ± SD |  |  |  |  |  |  |  |  |
| HbA1c, % | 8.68 ± 1.45 | 8.65 ± 1.32 | 8.65 ± 1.55 | 8.75 ± 1.48 | 0.004 | 0.065 | 0.069 | 0.069 |
| SBP, mmHg | 132.15 ± 15.69 | 132.35 ± 15.37 | 132.04 ± 16.24 | 132.05 ± 15.47 | 0.020 | 0.003 | 0.020 | 0.021 |
| DBP, mmHg | 76.93 ± 9.57 | 76.89 ± 9.78 | 76.87 ± 9.74 | 77.02 ± 9.19 | 0.002 | 0.016 | 0.014 | 0.016 |
| LDL-C, mmol/L | 2.43 ± 0.77 | 2.40 ± 0.71 | 2.40 ± 0.74 | 2.48 ± 0.84 | 0.002 | 0.096 | 0.097 | 0.097 |
| HDL-C, mmol/L | 1.13 ± 0.28 | 1.14 ± 0.28 | 1.14 ± 0.29 | 1.13 ± 0.26 | 0.001 | 0.027 | 0.028 | 0.028 |
| BMI, kg/m^2^ | 28.50 ± 4.01 | 28.45 ± 3.92 | 28.49 ± 4.15 | 28.57 ± 3.97 | 0.009 | 0.022 | 0.032 | 0.032 |
| Waist, cm | 96.52 ± 26.78 | 95.67 ± 9.73 | 96.55 ± 19.35 | 97.33 ± 40.79 | 0.061 | 0.028 | 0.076 | 0.076 |
| TC, mmol/L | 4.39 ± 0.94 | 4.36 ± 0.86 | 4.36 ± 0.92 | 4.45 ± 1.03 | 0.003 | 0.093 | 0.101 | 0.101 |
| Triglyceride, mmol/L | 1.89 ± 1.29 | 1.86 ± 1.26 | 1.87 ± 1.30 | 1.93 ± 1.30 | 0.005 | 0.048 | 0.054 | 0.054 |
| Creatinine (Serum), umol/L | 83.49 ± 37.37 | 83.28 ± 38.01 | 84.66 ± 41.91 | 82.56 ± 31.53 | 0.035 | 0.054 | 0.020 | 0.054 |
| eGFR, mL/min/1.73m^2^ | 86.95 ± 27.10 | 87.67 ± 27.69 | 87.93 ± 29.55 | 85.28 ± 23.72 | 0.009 | 0.095 | 0.089 | 0.095 |
| Fasting glucose, mmol/L | 9.64 ± 2.92 | 9.56 ± 2.73 | 9.58 ± 2.98 | 9.79 ± 3.02 | 0.006 | 0.072 | 0.084 | 0.084 |
|  |  |  |  |  |  |  |  |  |
| Prior severe hypoglycemia, % | 6.25% | 6.39% | 6.21% | 6.15% | 0.008 | 0.003 | 0.011 | 0.011 |
|  |  |  |  |  |  |  |  |  |
| Duration between first-line medication and third-line medication (year), Mean ± SD | 5.45 ± 2.82 | 5.51 ± 2.67 | 5.52 ± 2.75 | 5.33 ± 3.01 | 0.006 | 0.068 | 0.064 | 0.068 |
|  |  |  |  |  |  |  |  |  |

Supplemental Table 2. Baseline characteristics of patient with DPP4i, insulin or TZD as third-line medication after propensity score weighting (Cont.)

| Patient characteristics | After weighting | | | | | | | |
| --- | --- | --- | --- | --- | --- | --- | --- | --- |
|  |  | (A) | (B) | (C) | (A) vs (B) | (B) vs (C) | (A) vs (C) | Maximum pairwise ASMD |
|  | Total | DPP4i | Insulin | TZD | Pairwise ASMD | Pairwise ASMD | Pairwise ASMD |  |
| Duration of DM (year), Mean ± SD | 5.44 ± 2.97 | 5.42 ± 2.84 | 5.35 ± 2.91 | 5.54 ± 3.15 | 0.022 | 0.063 | 0.043 | 0.063 |
| Duration of DM (year), % |  |  |  |  | 0.002 | 0.080 | 0.078 | 0.080 |
| ≤5 years | 46.69% | 46.22% | 46.38% | 47.44% |  |  |  |  |
| 5-10 years | 46.19% | 48.17% | 48.46% | 42.02% |  |  |  |  |
| >10 years | 7.13% | 5.61% | 5.16% | 10.54% |  |  |  |  |
|  |  |  |  |  |  |  |  |  |
| Charlson Comorbidity Index (CCI), % |  |  |  |  | 0.045 | 0.045 | 0.045 | 0.045 |
| 1 or 2 | 16.39% | 15.85% | 16.18% | 17.13% |  |  |  |  |
| 3 | 26.84% | 26.54% | 26.54% | 27.41% |  |  |  |  |
| 4 | 25.54% | 26.07% | 26.05% | 24.53% |  |  |  |  |
| 5 | 15.21% | 15.58% | 14.95% | 15.12% |  |  |  |  |
| 6 or above | 16.01% | 15.96% | 16.28% | 15.80% |  |  |  |  |
|  |  |  |  |  |  |  |  |  |
| Note: * Imbalance covariate if pairwise ASMD >= 0.2 | | | | | |  |  |  |
| Abbreviation: ASMD = absolute standardized mean difference; SD = standard derivation; HbA1c = hemoglobin A1c; SBP = systolic blood pressure; DBP = diastolic blood pressure; LDL-C = low density lipoprotein - cholesterol; HDL-C = high density lipoprotein - cholesterol; BMI = body mass index; TC = total cholesterol; Urine ACR = urine albumin creatinine ratio; eGFR = estimated glomerular filtration rate; DM = diabetes mellitus | | | | | | | | |
