# Supplementary material for: Intensification with dipeptidyl peptidase-4 inhibitor, insulin, or thiazolidinediones and risks of all-cause mortality, cardiovascular diseases, and severe hypoglycemia in patients on metformin-sulfonylurea dual therapy: A retrospective cohort study
Source: PLoS Med. 2019 Dec 26;16(12):e1002999. doi: 10.1371/journal.pmed.1002999 (PMC6932752; doi:10.1371/journal.pmed.1002999)
Supplement: S3 Table — (DOCX) [file pmed.1002999.s004.docx]

| Supplemental Table 3. Hazard ratio of all-cause mortality, severe hypoglycemia, and cardiovascular disease events for comparisons amongst DPP4i, insulin, TZD and other glucose-lowering medications | | | | | | | | |
| --- | --- | --- | --- | --- | --- | --- | --- | --- |
|  |  |  |  |  |  |  |  |  |
|  | Reference | | | | | | | |
| **All-cause mortality** | DPP4i | | Insulin | | TZD | | Others | |
| DPP4i |  |  | HR | 0.440 | HR | 1.135 | HR | 0.776 |
|  |  |  | 95% CI | (0.397, 0.488) | 95% CI | (0.992, 1.298) | 95% CI | (0.695, 0.867) |
|  |  |  | P-value | <0.001* | P-value | 0.065 | P-value | <0.001* |
| Insulin | HR | 2.272 |  |  | HR | 2.578 | HR | 1.763 |
|  | 95% CI | (2.051, 2.516) |  |  | 95% CI | (2.304, 2.884) | 95% CI | (1.625, 1.913) |
|  | P-value | <0.001* |  |  | P-value | <0.001* | P-value | <0.001* |
| TZD | HR | 0.881 | HR | 0.388 |  |  | HR | 0.684 |
|  | 95% CI | (0.771, 1.008) | 95% CI | (0.347, 0.434) |  |  | 95% CI | (0.609, 0.769) |
|  | P-value | 0.065 | P-value | <0.001* |  |  | P-value | <0.001* |
| Others | HR | 1.288 | HR | 0.567 | HR | 1.462 |  |  |
|  | 95% CI | (1.154, 1.439) | 95% CI | (0.523, 0.615) | 95% CI | (1.301, 1.643) |  |  |
|  | P-value | <0.001* | P-value | <0.001* | P-value | <0.001* |  |  |
|  |  |  |  |  |  |  |  |  |
|  | Reference | | | | | | | |
| **Severe hypoglycemia** | DPP4i | | Insulin | | TZD | | Others | |
| DPP4i |  |  | HR | 0.653 | HR | 0.800 | HR | 0.414 |
|  |  |  | 95% CI | (0.586, 0.728) | 95% CI | (0.705, 0.909) | 95% CI | (0.374, 0.459) |
|  |  |  | P-value | <0.001* | P-value | <0.001* | P-value | <0.001* |
| Insulin | HR | 1.531 |  |  | HR | 1.225 | HR | 0.634 |
|  | 95% CI | (1.374, 1.707) |  |  | 95% CI | (1.095, 1.371) | 95% CI | (0.584, 0.689) |
|  | P-value | <0.001* |  |  | P-value | <0.001* | P-value | <0.001* |
| TZD | HR | 1.250 | HR | 0.816 |  |  | HR | 0.518 |
|  | 95% CI | (1.101, 1.419) | 95% CI | (0.729, 0.913) |  |  | 95% CI | (0.466, 0.575) |
|  | P-value | <0.001* | P-value | <0.001* |  |  | P-value | <0.001* |
| Others | HR | 2.414 | HR | 1.577 | HR | 1.932 |  |  |
|  | 95% CI | (2.180, 2.674) | 95% CI | (1.452, 1.712) | 95% CI | (1.739, 2.146) |  |  |
|  | P-value | <0.001* | P-value | <0.001* | P-value | <0.001* |  |  |
|  |  |  |  |  |  |  |  |  |
|  | Reference | | | | | | | |
| **Cardiovascular Diseases** | DPP4i | | Insulin | | TZD | | Others | |
| DPP4i |  |  | HR | 0.996 | HR | 1.030 | HR | 0.545 |
|  |  |  | 95% CI | (0.917, 1.081) | 95% CI | (0.938, 1.131) | 95% CI | (0.506, 0.588) |
|  |  |  | P-value | 0.925 | P-value | 0.549 | P-value | <0.001* |
| Insulin | HR | 1.004 |  |  | HR | 1.034 | HR | 0.548 |
|  | 95% CI | (0.925, 1.091) |  |  | 95% CI | (0.945, 1.132) | 95% CI | (0.511, 0.587) |
|  | P-value | 0.925 |  |  | P-value | 0.472 | P-value | <0.001* |
| TZD | HR | 0.971 | HR | 0.967 |  |  | HR | 0.529 |
|  | 95% CI | (0.884, 1.066) | 95% CI | (0.884, 1.058) |  |  | 95% CI | (0.488, 0.574) |
|  | P-value | 0.549 | P-value | 0.472 |  |  | P-value | <0.001* |
| Others | HR | 1.834 | HR | 1.826 | HR | 1.889 |  |  |
|  | 95% CI | (1.702, 1.977) | 95% CI | (1.704, 1.958) | 95% CI | (1.741, 2.050) |  |  |
|  | P-value | <0.001* | P-value | <0.001* | P-value | <0.001* |  |  |

Abbreviation: HR = hazard ratio; CI = Confidence interval
