# Supplementary material for: Intensification with dipeptidyl peptidase-4 inhibitor, insulin, or thiazolidinediones and risks of all-cause mortality, cardiovascular diseases, and severe hypoglycemia in patients on metformin-sulfonylurea dual therapy: A retrospective cohort study
Source: PLoS Med. 2019 Dec 26;16(12):e1002999. doi: 10.1371/journal.pmed.1002999 (PMC6932752; doi:10.1371/journal.pmed.1002999)
Supplement: S4 Table — (DOCX) [file pmed.1002999.s005.docx]

Supplemental Table 4. E-values for outcomes

|  | TZD (vs DPP4i) | | | | Insulin (vs TZD) | | | | Insulin (vs DPP4i) | | | |
| --- | --- | --- | --- | --- | --- | --- | --- | --- | --- | --- | --- | --- |
|  | HR | 95% CI | E-value | E-value CI | HR | 95% CI | E-value | E-value CI | HR | 95% CI | E-value | E-value CI |
| **All-cause mortality** | 0.888 | (0.776, 1.016) | 1.50 | 1.00 | 2.648 | (2.367, 2.963) | 4.74 | 4.17 | 2.352 | (2.123, 2.605) | 4.13 | 3.67 |
| **Severe hypoglycemia** | 1.249 | (1.099, 1.419) | 1.81 | 1.43 | 1.198 | (1.071, 1.340) | 1.69 | 1.35 | 1.496 | (1.342, 1.668) | 2.36 | 2.02 |
| **Cardiovascular Diseases** | 1.005 | (0.915, 1.104) | 1.07 | 1.00 | 0.965 | (0.883, 1.056) | 1.23 | 1.00 | 0.970 | (0.893, 1.053) | 1.21 | 1.00 |
|  |  |  |  |  |  |  |  |  |  |  |  |  |
| Abbreviation: HR = hazard ratio; CI = Confidence interval | | | |  |  |  |  |  |  |  |  |  |
