# Supplementary material for: Intensification with dipeptidyl peptidase-4 inhibitor, insulin, or thiazolidinediones and risks of all-cause mortality, cardiovascular diseases, and severe hypoglycemia in patients on metformin-sulfonylurea dual therapy: A retrospective cohort study
Source: PLoS Med. 2019 Dec 26;16(12):e1002999. doi: 10.1371/journal.pmed.1002999 (PMC6932752; doi:10.1371/journal.pmed.1002999)
Supplement: S5 Table — (DOCX) [file pmed.1002999.s006.docx]

| Supplemental Table 5. Adjusted hazard ratios for the associations between the outcomes and observed baseline covariates | | | |
| --- | --- | --- | --- |
|  |  |  |  |
|  | Adjusted hazard ratio (95% CI) | | |
| Baseline covariates | **All-cause mortality** | **Severe hypoglycemia** | **Cardiovascular Diseases** |
| Gender | 1.145 (1.029, 1.273) | 0.958 (0.837, 1.095) | 1.357 (1.223, 1.505) |
| BMI | 0.919 (0.905, 0.933) | 1.005 (0.989, 1.021) | 1.036 (1.023, 1.049) |
| HbA1c | 0.781 (0.747, 0.817) | 1.089 (1.037, 1.143) | 1.041 (1.003, 1.081) |
| SBP | 1.002 (0.998, 1.005) | 0.998 (0.993, 1.002) | 1.000 (0.997, 1.003) |
| DBP | 0.987 (0.981, 0.993) | 1.001 (0.993, 1.008) | 1.011 (1.005, 1.017) |
| LDL-C | 1.084 (0.827, 1.421) | 1.023 (0.772, 1.357) | 0.910 (0.685, 1.211) |
| total cholesterol | 0.812 (0.624, 1.057) | 1.155 (0.877, 1.522) | 1.240 (0.937, 1.642) |
| HDL-C | 1.218 (0.899, 1.650) | 1.060 (0.746, 1.505) | 0.977 (0.707, 1.350) |
| serum creatinine | 1.001 (1.000, 1.002) | 1.004 (1.003, 1.005) | 1.000 (0.999, 1.002) |
| triglyceride | 1.005 (0.898, 1.125) | 1.013 (0.906, 1.132) | 0.921 (0.816, 1.040) |
| fasting glucose | 0.991 (0.969, 1.013) | 1.012 (0.987, 1.037) | 1.007 (0.987, 1.028) |
| Duration between first-line medication and third-line medication | 0.944 (0.910, 0.980) | 1.005 (0.962, 1.050) | 0.955 (0.923, 0.989) |
| Duration of DM | 1.096 (1.058, 1.135) | 0.931 (0.894, 0.970) | 1.060 (1.026, 1.095) |
| CCI | 1.545 (1.515, 1.576) | 1.386 (1.348, 1.426) | 1.761 (1.726, 1.797) |
| Abbreviation: CI = confidence interval; BMI = body mass index; HbA1c = hemoglobin A1c; SBP = systolic blood pressure; | | | |
| DBP = diastolic blood pressure; LDL-C = low density lipoprotein - cholesterol; HDL-C = high density lipoprotein - cholesterol; | | | |
| DM = diabetes mellitus; CCI = Charlson comorbidity index | | | |
