# Supplementary material for: Intensification with dipeptidyl peptidase-4 inhibitor, insulin, or thiazolidinediones and risks of all-cause mortality, cardiovascular diseases, and severe hypoglycemia in patients on metformin-sulfonylurea dual therapy: A retrospective cohort study
Source: PLoS Med. 2019 Dec 26;16(12):e1002999. doi: 10.1371/journal.pmed.1002999 (PMC6932752; doi:10.1371/journal.pmed.1002999)
Supplement: S6 Table — (DOCX) [file pmed.1002999.s007.docx]

| Supplemental Table 6. Hazard ratio of all-cause mortality, severe hypoglycemia, and cardiovascular disease events using complete case analysis | | | | | | | | |  |
| --- | --- | --- | --- | --- | --- | --- | --- | --- | --- |
| Event | TZD (vs DPP4i) | | | Insulin (vs TZD) | | | Insulin (vs DPP4i) | | |
|  | HR | 95% CI | P-value | HR | 95% CI | P-value | HR | 95% CI | P-value |
| **All-cause mortality** | 0.673 | (0.515, 0.879) | 0.004* | 5.090 | (3.925, 6.602) | <0.001* | 3.425 | (3.081, 3.807) | <0.001* |
| **Severe hypoglycemia** | 0.799 | (0.607, 1.051) | 0.109 | 2.399 | (1.826, 3.153) | <0.001* | 1.917 | (1.702, 2.159) | <0.001* |
| **Cardiovascular Diseases** | 0.690 | (0.567, 0.840) | <0.001* | 1.969 | (1.614, 2.402) | <0.001* | 1.359 | (1.242, 1.487) | <0.001* |

Abbreviation: HR = hazard ratio; CI = Confidence interval
